# Survey of Anticoagulation Practices with the Impella Percutaneous Ventricular Assist Device at High-Volume Centers

**DOI:** 10.1155/2019/3791307

**Published:** 2019-03-04

**Authors:** Brent N. Reed, Robert J. DiDomenico, J. Erin Allender, James C. Coons, Jenna F. Cox, Daniel Johnson, Carrie S. Oliphant, Douglas L. Jennings

**Affiliations:** ^1^University of Maryland School of Pharmacy, USA; ^2^University of Illinois at Chicago, USA; ^3^WakeMed Health & Hospitals, USA; ^4^University of Pittsburgh School of Pharmacy and UPMC Presbyterian Hospital, USA; ^5^Palmetto Health Richland, USA; ^6^Vanderbilt University Medical Center, USA; ^7^Methodist Healthcare-University Hospital, USA; ^8^New York Presbyterian Columbia University Medical Center, USA

## Abstract

**Objectives:**

To characterize anticoagulation practices with the Impella percutaneous ventricular assist device (pVAD).

**Background:**

Managing anticoagulation in patients being supported by the Impella pVAD is made challenging by several unique features of the device. These include the release of a dextrose-based purge solution containing unfractionated heparin (UFH), the need to concurrently administer systemic anticoagulation with intravenous UFH, and the lack of an alternative strategy in patients with contraindications to UFH.

**Methods:**

To characterize anticoagulation practices with the Impella pVAD, we conducted a survey of centers in the United States performing a high volume of Impella cases, which we defined as > 1 per month. Centers were contacted via email or phone and individuals who agreed to participate were provided with a link to complete the survey online. The primary measures of interest were variations in practice across centers and variations from the manufacturer's recommendations.

**Results:**

Practices varied considerably among respondents (65 of 182 centers, or 35.7%) and often diverged from manufacturer recommendations. Approximately half of centers (52.4%) reported using a UFH concentration of 50 units/mL in the purge solution, whereas most of the remaining centers (41.3%) reported using lower concentrations. Strategies for the initiation and adjustment of systemic therapy also varied, as did practices for routinely monitoring for hemolysis. Nearly one-fifth of centers (16.7%) had not developed an alternative strategy for the purge solution in patients with contraindications to UFH. Most centers (58.4%) reported using argatroban or bivalirudin in this scenario, a strategy that diverges from the manufacturer's recommendations.

**Conclusions:**

Given these findings, studies to determine a systematic approach to anticoagulation with the Impella device are warranted.

## 1. Introduction

Percutaneous ventricular assist devices (pVADs) such as the Impella series (Abiomed; Danvers, MA) may be used for short-term mechanical circulatory support (MCS) during high-risk procedures, or as a bridge to recovery or advanced therapies (e.g., durable MCS) in patients with cardiogenic shock [1, 2]. Compared to intra-aortic balloon pump (IABP) counterpulsation, Impella devices improve intracardiac hemodynamics but these differences have not translated into improvements in long-term morbidity and mortality [3–5].

**Figure 1:**
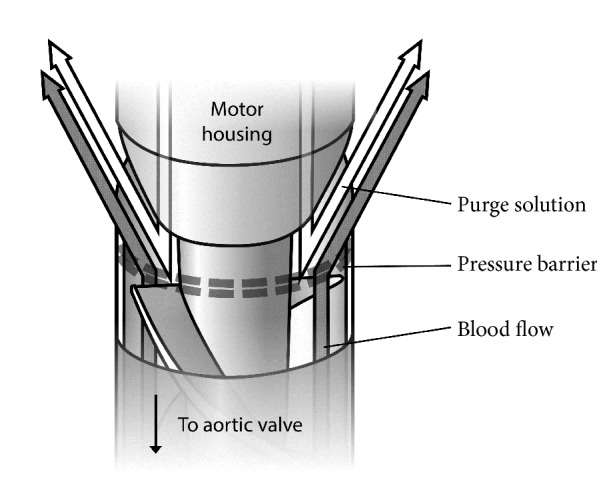
*Impella Outlet Area. *The Impella purge solution is released from the motor housing in a countercurrent direction to the flow of blood being expelled from the left ventricle. The resulting pressure barrier prevents blood from entering the motor housing (image created by the authors).

One unique facet of Impella devices is the release of a dextrose-based purge solution from the motor housing (Figure 1). The purge solution flows countercurrently to blood flow, creating a pressure barrier to prevent blood from entering the motor housing. To further reduce the risk of device thrombosis, the manufacturer also recommends that the purge solution contain 50 units/mL of unfractionated heparin (UFH). Since flow rates of the purge solution are automatically regulated by the device to maintain a specific pressure range (300-1100 mm Hg), UFH exposure may vary considerably in a 24-hour period [6, 7].

Distribution of UFH to the peripheral circulation also results in systemic drug exposure [8]. However, the extent to which the purge solution contributes to systemic anticoagulation can vary, thus concurrent administration of intravenous (IV) UFH is also recommended (goal activated clotting time of 160-180 seconds) [6]. Since the default concentration for most IV UFH products is 100 units/mL, patients are often exposed to two sources of UFH in different concentrations, via different routes of administration (i.e., purge and systemic), and at different infusion rates.

Altogether these factors make the management of anticoagulation during Impella support a considerable challenge. Minimal guidance is provided in the literature, as the anticoagulation practices used in clinical trials have either varied or have not been clearly reported [8]. Also lacking are recommendations in patients with contraindications to UFH such as heparin-induced thrombocytopenia (HIT). Despite an increased risk of thrombosis with HIT, alternative anticoagulants have not been studied, and a dextrose-only solution is recommended instead [6].

Given the complexity of these issues, we hypothesized that significant variability in anticoagulation practices would exist among Impella centers, and we sought to characterize this via a nationwide survey.

## 2. Methods

We contacted the manufacturer to obtain a list of all centers in the United States who currently use Impella devices. To limit the potentially confounding effect of institutional experience with the device, we limited the survey to high-volume centers, which we defined* a priori* as those performing > 1 Impella case per month. After filtering the list by volume, we contacted all centers performing an average of > 1 case per month via email and/or phone. Our outreach correspondence specified that individuals participating in the survey would be representing their center and responses should reflect the practices specified by institutional guidelines, or those used most commonly in practice. Individuals who agreed to participate in the research were provided with a link to the survey, which was administered online using the Qualtrics® platform (Provo, UT). Participation in the survey was voluntary and responses remained anonymous. The study was deemed exempt by the local institutional review board.

Data collected in the survey included institution size (number of beds), case volume by model, indications for Impella use, purge solution characteristics, and anticoagulation practices (e.g., initiation and adjustment, monitoring, alternatives in patients with contraindications to UFH). The survey consisted primarily of multiple-choice questions with several opportunities for write-in responses (see the Supplementary Appendix for survey questions (Available here)). The survey was developed collaboratively by the authors and underwent several revisions for accuracy and clarity based on physician, pharmacist, and nurse feedback. A pilot version of the survey was also distributed via email to a network of clinical pharmacists, and no additional revisions were suggested.

The primary measures of interest in our study were how practices varied across centers as well as variations from the manufacturer's recommendations. These were analyzed using descriptive statistics and are reported as totals and proportions. An exploratory inferential analysis was also conducted, in which the highest volume centers (i.e., those performing the median number of cases or higher) were compared to the remaining centers using t-tests and chi-square or Fisher's exact test as appropriate. All analyses were performed in SPSS version 23 (IBM Corp; Armonk, NY).

## 3. Results

A total of 182 centers met our inclusion criterion, of which 65 (35.7%) responded to some or all of the survey questions. Of these, 56 (86.2%) responded to all survey questions, and most questions were answered by ≥ 60 centers. The mean size of participating institutions was 606 ± 241 beds and the median number of cases/month was 7 (interquartile range 4-11). The Impella 2.5 was used most frequently (76.9% of centers), followed by the CP and 5.0 models (63.1% and 61.5%, respectively) (Table 1). Less than half of centers (43.1%) reported using the right-sided Impella RP. By far the most common indications for Impella support were acute decompensated heart failure/cardiogenic shock and high-risk percutaneous coronary intervention (90.8% and 81.5%, respectively). A majority of centers (83.1%) reported using D5W as their default purge concentration and an additional 10.8% planned to do so. Of the nine centers that were not using D5W, only four used D20W, the concentration previously recommended by the manufacturer.

**Figure 2:**
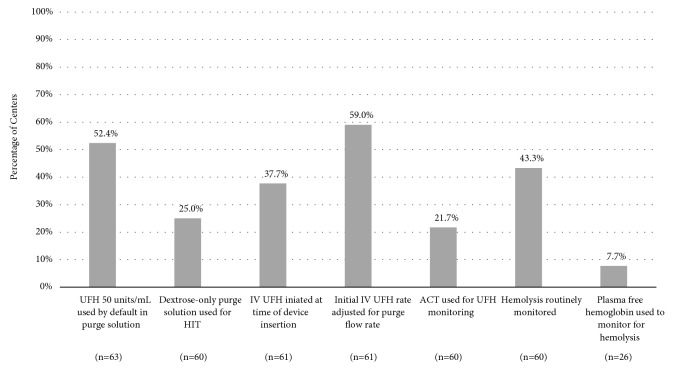
*Summary of Impella Anticoagulation Practices*. No consensus emerged with regard to any of the practices queried in the survey. A slight majority of centers used the default concentration of UFH recommended by the manufacturer and adjusted the initial IV UFH rate for purge flow. Many of the above practices diverge from the manufacturers' recommendations. Abbreviations: ACT: activated clotting time, IV: intravenous, and UFH: unfractionated heparin.

Anticoagulation practices varied significantly among the centers we surveyed (Table 2; summarized in Figure 2). Regarding the purge solution, approximately half of centers (52.4%) reported using the UFH concentration recommended by the manufacturer (50 units/mL) [6]. Altogether, approximately 41% reported using lower concentrations (12.5 to 25 units/mL), and a minority of centers (4.8%) reported using dextrose-only solutions. Nearly one-fifth (16.7%) of centers had not yet developed an alternative strategy for the purge solution in patients with a contraindication to UFH. Only 25% reported using an anticoagulant-free (i.e., dextrose only) purge solution for HIT as recommended by the manufacturer [6]. Over half (58.4%) reported using a purge solution containing argatroban, bivalirudin, or either in this scenario. The remainder of centers (16.7%) had not yet developed a strategy for HIT.

Practices also varied with regard to the use of IV UFH and monitoring. No consensus emerged regarding whether IV UFH should be initiated at the point of device insertion (37.7%) or only after patients are subtherapeutic on the purge infusion alone (49.1%). A minority (18.0%) of centers initiated UFH with a bolus, and most (59.0%) adjusted the initial IV UFH infusion for the current purge flow rate. Activated partial thromboplastin time (aPTT) was the parameter most commonly used to monitor UFH (56.7%) rather than activated clotting time (ACT, 21.7%) as recommended by the manufacturer.

Fewer than half of centers (43.4%) reported routinely monitoring for hemolysis during Impella support. Of those who did, lactate dehydrogenase (LDH) was reported as being the most common parameter used for this purpose (100%). Only 2 of 26 centers (7.7%) reported using plasma-free hemoglobin (pfHgb) to monitor for hemolysis as suggested by the manufacturer. In our exploratory inferential analysis comparing the highest volume centers to all others, the former were more likely to routinely monitor for hemolysis (21 of 43 vs. 5 of 22, p=0.043). No other differences were noted between the two groups.

## 4. Discussion

To our knowledge, this is the first study to characterize real-world anticoagulation practices with the Impella pVAD. Consistent with our expectations, we found that practices varied considerably, even among this cohort of high-volume centers. This is not surprising given that the anticoagulation strategies used in clinical trials are often unclear or have varied across studies [8]. For example, in the ISAR-SHOCK trial comparing the Impella device to IABP in patients with cardiogenic shock after myocardial infarction, a dextrose-only purge solution was used and IV UFH was titrated to target an aPTT of 60-80 seconds [3]. However, in the PROTECT-II trial comparing the two devices during high-risk percutaneous coronary intervention (PCI), either UFH or bivalirudin could be used for systemic anticoagulation and no information was provided regarding the content of the purge solution [4].

The number of centers whose strategies diverged from the manufacturers' recommendations was noteworthy. Regarding the use of lower UFH concentrations in the purge solution, we attributed this to the manufacturer's preference for D5W rather than D20W as the default fluid for the purge solution beginning in September 2015 [9]. Given the lower viscosity of D5W compared to D20W, 30-40% higher flow rates are to be expected, resulting in greater systemic exposure to UFH via the purge solution. The effect of this change in practice was recently evaluated in a case series of 12 patients, in which a higher number of supratherapeutic aPTT values (5 of 8) and bleeding events (3 of 8) were observed in patients receiving a purge solution comprised of UFH 50 units/mL diluted in a D5W solution compared to those receiving it in a D20W solution; no supratherapeutic aPTTs or bleeding events were observed in the latter group [10].

Other deviations from the manufacturer's recommendations likely reflect local standards of practice. For example, the use of aPTT to monitor systemic anticoagulation in patients requiring prolonged Impella support likely reflects the preference for this parameter to monitor UFH in other indications and the lack of widespread availability of ACT monitoring outside of the catheterization lab. The feasibility of using an aPTT-based nomogram to monitor and adjust UFH therapy in this population has been previously reported [7]. At least one report has also suggested that a nomogram based on anti-Xa monitoring may also be useful, with therapeutic ranges of 0.15-0.25 units/mL and 0.20-0.30 units/mL for low and high levels of anti-Xa activity, respectively [11]. Although monitoring and adjustment of anticoagulation therapy should be standardized, the most optimal parameter to use for this practice is currently unknown and should be the subject of future research.

Although a notable number of centers had not developed a strategy for patients with HIT, over half reported using argatroban or bivalirudin in the purge solution, which deviates from the manufacturer's recommendation to use a dextrose-only solution [6]. This finding was unsurprising given the hypothesized rationale for placing an anticoagulant in the purge solution (i.e., to prevent device thrombosis), the risk of which would be presumably higher in patients with hypercoagulable states like HIT. Of the available alternatives to UFH in patients with HIT, at least three cases using argatroban in the Impella purge solution have been reported [12, 13].

Finally, hemolysis is a well-recognized complication of the Impella device, and cumulative rates of up to 62.5% have been reported [14]. The manufacturer recommends monitoring for hemolysis and specifically the use of pfHgb, although details regarding frequency or duration are not provided [6]. Monitoring for this complication has important implications for anticoagulation therapy, as decreases in serum hemoglobin due to undetected hemolysis may prompt inappropriate changes in anticoagulation strategy due to concerns for bleeding (e.g., premature discontinuation of UFH). Only 43.3% of centers reported routinely monitoring for this complication, although it appeared to be more frequent among centers with the highest volume. Of the centers that routinely monitored for hemolysis, all monitored LDH whereas only 7.7% reported also monitoring pfHgb. Recently, increases in pfHgb but not LDH were shown to predict the development of hemolysis in patients being supported by Impella devices, suggesting that the appropriate monitoring parameter for this complication should also be the subject of future research [15].

The main limitation of our study is that it only represents a subset of Impella centers. The results may thus be subject to selection bias, as those who felt strongly about anticoagulation practices with the device may have been more likely to respond. However, we expect this risk to be low given the diversity (in terms of both size and geographic region) of the centers represented in the survey. Although some may consider a response rate of 35.7% as being low, it is comparable to the mean expected response rate of Internet-based surveys according to meta-analytic research (39.6%) [16]. Another limitation is that we did not collect the role or training background of survey respondents, which may have introduced interrater variability into our results. We attempted to control for this by requesting that individuals only participate if they were familiar with their institution's guidelines or standards of practice or to recommend an alternative individual who was. Finally, we did not survey centers on their rates of thrombosis or bleeding; thus we are unable to connect the variability in anticoagulation practices with clinical outcomes. However, we believe this would have made our survey too cumbersome to complete and would have lowered our response rate considerably.

In summary, we feel that the significant variability in anticoagulation practices observed in our study warrants further investigation, particularly given the baseline risks of thrombosis and bleeding in patients receiving Impella support.

## Figures and Tables

**Table 1:** 

Characteristics of Study Centers	Centers
Impella devices used (n=65)	
2.5	50 (76.9%)
5.0	40 (61.5%)
CP	41 (63.1%)
RP	28 (43.1%)

Indications for Impella use (n=65)	
Acute decompensated heart failure/cardiogenic shock	59 (90.8%)
High-risk percutaneous intervention	53 (81.5%)
Acute coronary syndromes	39 (60.0%)
Cardiac arrest	20 (30.8%)
Other ventricular arrhythmias	14 (21.5%)
Other	3 (4.6%)

**Table 2:** 

Anticoagulation Practice	Centers (%)
Default purge heparin concentrations (n=63)	
0 units/mL	3 (4.8%)
12.5 units/mL	8 (12.7%)
25 units/mL	18 (28.6%)
50 units/mL	33 (52.4%)
Other	1 (1.6%)

Alternative purge in patients with a contraindication to heparin (n=60)	
Remove anticoagulant from purge	15 (25.0%)
Argatroban	22 (36.7%)
Bivalirudin	6 (10.0%)
Either argatroban or bivalirudin	7 (11.7%)
No strategy developed for this scenario	10 (16.7%)

Timing of intravenous heparin initiation (n=61)	
At device insertion, prior to assessing anticoagulation status	23 (37.7%)
Only after the patient is subtherapeutic on the purge solution alone	30 (49.2%)
Other	8 (13.1%)

Bolus of heparin administered during intravenous administration (n=61)	11 (18.0%)

Intravenous heparin adjusted for initial purge flow (n=61)	36 (59.0%)

Default heparin monitoring (n=60)	
Activated partial thromboplastin time	34 (56.7%)
Anti-Xa concentration	8 (13.3%)
Activated clotting time	13 (21.7%)
Other	5 (8.3%)

Monitoring for hemolysis is performed routinely (n=60)	26 (43.3%)

Hemolysis laboratories monitored (n=26)	
Lactate dehydrogenase	26 (100.0%)
Indirect bilirubin	13 (50.0%)
Serum haptoglobin	10 (38.5%)
Plasma free hemoglobin	2 (7.7%)

## Data Availability

The data used to support the findings of this study are included within the article.

## Supplementary Materials

Supplementary Materials A copy of the survey used in this analysis has been provided as supplementary material.
