# Supplementary material for: Survey of Anticoagulation Practices with the Impella Percutaneous Ventricular Assist Device at High-Volume Centers
Source: J Interv Cardiol. 2019 Mar 4;2019:3791307. doi: 10.1155/2019/3791307 (PMC6739784; doi:10.1155/2019/3791307)
Supplement: Supplementary Materials — A copy of the survey used in this analysis has been provided as supplementary material. [file 3791307.f1.pdf]

Supplementary Appendix

Below is a copy of the survey used for this analysis.

Introduction:
An optimal anticoagulation strategy for patients receiving mechanical circulatory support with the Impella percutaneous ventricular assist device is currently unknown. The purpose of this survey is to characterize current anticoagulation practices, which may then be used to help shape future management strategies. Your participation in this survey is entirely voluntary. It should take approximately 8 minutes to complete.

The following questions refer to the indications for which the Impella device is used at your facility, and an estimate of your current caseload.

1. For which of the following indications do you use Impella devices at your facility? Please select all that apply.
    a. Acute coronary syndromes
    b. High-risk percutaneous coronary intervention
    c. Acute decompensated heart failure / cardiogenic shock
    d. Cardiac arrest
    e. Other ventricular arrhythmias
    f. Other (please describe)

2. Which of the following Impella models are used at your facility? Please select all that apply.
    a. Impella 2.5
    b. Impella 5.0
    c. Impella CP
    d. Impella RP

[Questions 3-6 are shown based on options selected in Question #2]

3. Approximately how many cases are performed with the **Impella 2.5** at your facility per month?
    a. 0 to 2
    b. 3 to 5
    c. 6 to 10
    d. > 10

4. Approximately how many cases are performed with the **Impella 5.0** at your facility per month?
    a. 0 to 2
    b. 3 to 5
    c. 6 to 10
    d. > 10

5. Approximately how many cases are performed with the **Impella CP** at your facility per month?
    a. 0 to 2
    b. 3 to 5
    c. 6 to 10
    d. > 10

6. Approximately how many cases are performed with the **Impella RP** at your facility per month?
    a. 0 to 2
    b. 3 to 5
    c. 6 to 10
    d. > 10

The following questions refer to the Impella purge solution at your facility.

7. Abiomed recently issued communication recommending that D5W be used as the default purge solution concentration. Which of the following is true regarding the concentration at your facility?
    a. We converted to D5W once we were aware of the updated recommendation.
    b. We were already using D5W as our default concentration prior to the updated recommendation.
    c. We have not yet converted to D5W but are planning to.
    d. We do not plan on converting to D5W in the foreseeable future.
    e. We were not aware of this recommendation.

[If c, d, or e is selected in #7 above, Question #8 is shown]

8. What is the default dextrose concentration at your facility?
    a. D10W
    b. D20W
    c. D50W
    d. Other (please describe)

9. Which of the following dextrose concentrations may be used at your facility if necessary to maintain goal purge pressure? Please select all that apply.
    a. D5W
    b. D10W
    c. D20W
    d. D50W
    e. Other (please describe)

10. What is the default heparin concentration used in the purge solution at your facility?
    a. 0 units/mL (no heparin)
    b. 12.5 units/mL
    c. 25 units/mL

d. 50 units/mL
e. 100 units/mL
f. Other (please describe)

11. Which of the following heparin concentrations can be used in the purge solution at your facility if necessary? Please select all that apply.
    a. 0 units/mL (no heparin)
    b. 12.5 units/mL
    c. 25 units/mL
    d. 50 units/mL
    e. 100 units/mL
    f. Other (please describe)

The following questions refer to systemic anticoagulation in patients receiving Impella support at your facility.

12. When do you initiate a systemic heparin infusion with the Impella device at your facility?
    a. At the time of device insertion, prior to obtaining anticoagulation parameters on the purge solution alone.
    b. Only after it is known that the patient's anticoagulation parameters are sub-therapeutic on the purge solution alone.
    c. Other (please describe).

13. When you initiate a systemic heparin infusion at your facility, what is your initial bolus dose of heparin? In your answer, please indicate whether the dose is in units/kg or units. Enter "0" if no bolus is administered. *[Text box for response]*

14. Is the initial infusion rate of heparin at your facility adjusted for purge flow rate?
    a. Yes
    b. No

[If a is selected above, Question #15 displays.]

15. Please describe how you adjust the initial infusion rate based on purge flow rate at your facility. *[Text box for response]*

[If b is selected above, Question #16 displays.]

16. What is the default initial infusion rate at your facility? In your answer, please indicate whether the rate is in units/kg/hour or units/hour. *[Text box for response]*

17. What maximum initial infusion rate of heparin is used at your facility?
    a. We do not have a maximum initial infusion rate.
    b. We cap at a specific infusion rate (please describe) *[Text box]*
    c. We cap at a specific body weight (please describe) *[Text box]*

18. What parameter is used to monitor heparin in patients receiving extended Impella support (i.e., post-procedure) at your facility?
    a. Activated partial thromboplastin time (aPTT)
    b. Anti-Xa concentration
    c. Activated Clotting Time (ACT)
    d. Other (please describe)

The following questions refer to the use of alternative anticoagulants with the Impella device.

19. What anticoagulant is used in the <u>purge solution</u> for patients with heparin-induced thrombocytopenia receiving Impella support at your facility?
    a. None (i.e., dextrose-only solution)
    b. Argatroban
    c. Bivalirudin
    d. Either argatroban or bivalirudin may be used
    e. We have not yet developed a strategy for this scenario

20. What anticoagulant is used for <u>systemic anticoagulation</u> for patients with heparin-induced thrombocytopenia receiving Impella support at your facility?
    a. Argatroban
    b. Bivalirudin
    c. Either argatroban or bivalirudin may be used
    d. We do not yet developed a strategy for this scenario

[If argatroban, bivalirudin, or either is selected, Question #21 is shown]

21. Does your facility have an initial infusion rate specifically designated for patients receiving Impella support?
    a. Yes
    b. No

[page break]

22. Does your facility routinely monitor hemolysis laboratories, such as lactate dehydrogenase (LDH), in patients receiving Impella support?
    a. Yes
    b. No

[If <u>Yes</u> is selected in #22, Question #23 shown]

23. Which of the following hemolysis laboratories are routinely monitored? Please select all that apply.
    a. Lactate dehydrogenase (LDH)
    b. Serum haptoglobin
    c. Indirect bilirubin
    d. Other (please describe)

The following questions refer to the facility where you provide care for patients receiving Impella support.

24. What is the name of the facility? *[Text box]*

25. Where is the facility located? *[Text boxes for city, state/territory, country]*

26. What is the approximate number of beds at your facility? *[Text box for number of beds]*
